# Beyond the HLA Genes in Gluten-Related Disorders

**DOI:** 10.3389/fnut.2020.575844

**Published:** 2020-11-12

**Authors:** Michele Sallese, Loris Riccardo Lopetuso, Konstantinos Efthymakis, Matteo Neri

**Affiliations:** ^1^Department of Medical, Oral and Biotechnological Sciences, ‘G. D'Annunzio' University of Chieti–Pescara, Chieti, Italy; ^2^Center for Advanced Studies and Technology (CAST), ‘G. D'Annunzio' University of Chieti-Pescara, Chieti, Italy; ^3^Department of Medicine and Aging Sciences, ‘G. D'Annunzio' University of Chieti–Pescara, Chieti, Italy

**Keywords:** HLA, DQ2 and DQ8, non-HLA genes, gene expression, non-celiac gluten sensitivity (NCGS)

## Abstract

Most common food grains contain gluten proteins and can cause adverse medical conditions generally known as gluten-related disorders. Celiac disease is an immune-mediated enteropathy triggered by gluten in individuals carrying a specific genetic make-up. The presence of the human leukocyte antigens (HLA)-DQ2 and HLA-DQ8 haplotypes together with gluten intake is a necessary, although not sufficient, condition, to develop celiac disease. Fine mapping of the human genome has revealed numerous genetic variants important in the development of this disease. Most of the genetic variants are small nucleotide polymorphisms located within promoters and transcriptional enhancer sequences. Their importance is underlined by an increased risk in DQ2/DQ8 carriers who also have these non-HLA alleles. In addition, several immune-mediated diseases share susceptibility loci with celiac disease, shedding light on the reasons for co-occurrence between these diseases. Finally, most of the genes potentially involved in celiac disease by fine genetic mapping of non-HLA loci were confirmed in gene expression studies. In contrast to celiac disease, very little is known about the genetic make-up of non-celiac wheat sensitivity (NCWS), a clinically defined pathology that shares symptoms and gluten dependence with the celiac disease. We recently identified differentially expressed genes and miRNAs in the intestinal mucosa of these patients. Remarkably, the differentially expressed genes were long non-coding RNAs possibly involved in the regulation of cell functions. Thus, we can speculate that important aspects of these diseases depend on alteration of regulatory genetic circuits. Furthermore, our finding suggests that innate immune response is involved in the pathogenic mechanism of NCWS. This review is intended to convey the idea that in order to fully understand celiac disease and its relationship with other gluten-related disorders, it is worth learning more about non-HLA variants.

## Introduction

Celiac disease is an immune-mediated enteropathy triggered by ingestion of gluten in genetically predisposed individuals (1, 2). Gluten is a protein mixture of prolamins and glutenin present in most diffused food cereals like wheat, rye, and barley. Gluten proteins are incompletely fragmented by the digestive proteases and residual peptides, crossing the intestinal epithelial barrier, reach the lamina propria (3, 4). Gluten peptides have cytotoxic, immunomodulatory and permeabilizing effects/properties. Contact with enterocytes leads to the production of interleukin (IL)15, a key mediator of celiac disease, the release of zonulin, and the activation of tissue transglutaminase (TG2) (5, 6). IL15 upregulates human leukocyte antigen (HLA)-E and major histocompatibility complex class I chain-related protein A (MICA) on enterocytes and their corresponding receptors, as well as cluster of differentiation (CD)94 and natural killer group 2D (NKG2D) on natural killer and CD8^+^ lymphocytes, thus contributing to mucosal damage (6, 7). Zonulin leads to the loosening of tight junctions and increased intestinal permeability. Glutamine residues of gluten peptides in the lamina propria are deamidated to glutamic acid by activated tissue transglutaminase (TG2). The introduction of negative charges into gluten peptides promotes their stable interaction with HLA class II in antigen presenting cells (APC) and thus the presentation of this antigens to T lymphocytes in lamina propria (8–10). Activated CD4^+^ T-cells produce IL21 and interferon (IFN)γ that support the activation of intraepithelial CD8^+^ lymphocytes which in turn damage enterocytes and cause villous atrophy (6). Cytokines released by activated T cells also contribute to the differentiation of B lymphocytes into plasma cells and the production of antibodies, mainly of the IgA subtype, against gluten peptides and TG2. The presence of anti-TG2 autoantibodies and villous atrophy, two main features of celiac disease, are also essential diagnostic markers (1).

The onset of celiac disease, besides gluten ingestion and the expression of specific HLAs, requires additional ill-defined factors. For example, a particular stressful event like the loss of a relative, a pregnancy, or an infection have been considered potential triggers of celiac disease. Nevertheless, even the simultaneous presence of these conditions not necessarily leads to the development of the disease, likely because additional genetic factors are involved. In this context, genetic factors beyond HLA have gained consistent relevance either as predisposing to the occurrence of celiac disease or as favoring the progression toward complicated disease or the presence of extraintestinal or associated diseases.

Moreover, some patients claiming persistent symptoms related to gluten consumption including abdominal distension and pain, diarrhea, tiredness, and dermatitis show negative celiac disease serology and preserved mucosal architecture. This characteristic phenotype has been defined non-celiac gluten sensitivity (NCWS), as symptoms respond to gluten exclusion (11, 12). Patients with NCWS have a normal intestinal mucosa on histological examination, and intraepithelial lymphocytes (IELs) counts are <25 per 100 enterocytes in about 60% of cases while ranging from 25 to 40 in the rest of cases. To date, very little is known about the pathological mechanisms, environmental factors, and genetic makeup predisposing to NCWS.

In this minireview we report the current knowledge regarding genetic variants and gene expression changes that have been associated to celiac disease and NCWS, focusing on non-HLA genes.

## HLA Locus is the Main Predisposing Factor For the Development of Celiac Disease

**Figure 1:**
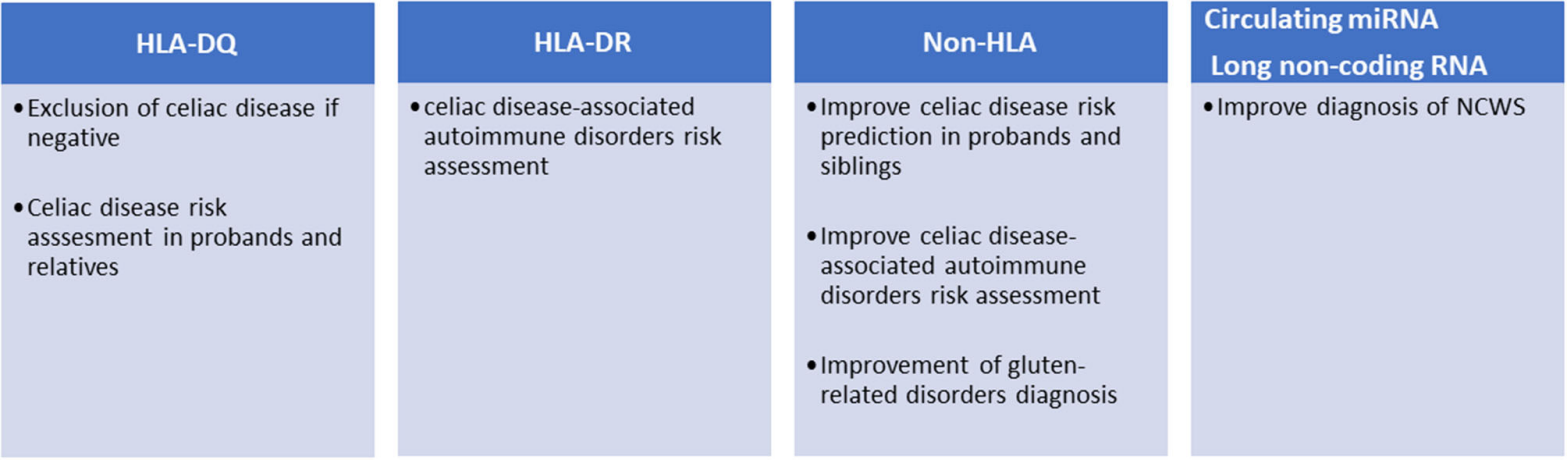
Current and potential clinical applications of genetic markers in gluten-related disorders.

More than 90% of patients affected by celiac disease are carriers of HLA-DQ2, while the rest are carriers of HLA-DQ8 (13). The expression of these HLAs appears a necessary condition for celiac disease development, while their absence almost definitively excludes it (Figure 1). The presence of HLA-DQ2/DQ8 haplotypes is often used to settle borderline serological and histological tests. The prevalence of HLA-DQ2/DQ8 in the general population ranges from 30 to 40%, but only about 3% of the carriers develop celiac disease suggesting that additional genetic factors may be required (14). HLA-DQ2 is a heterodimer encoded by different DQA1 (α chain) and DQB1 (β chain) genes. Specifically, DQA1^*^0501 and DQB1^*^0201 form the HLA-DQ2.5, while the DQA1^*^0201 and DQB1^*^0202 form the HLA-DQ2.2. The DQ2.5 haplotype can be carried in *cis*-configuration, where DQA1^*^0501 and DQB1^*^0201 are both present on the same chromosome copy (paternal, maternal, or both); alternatively, it can be present in *trans*-configuration where the genes for the α and β chains are each separately present on the parental chromosomes. Haplotypes can further be homozygous or heterozygous; thus, HLA-DQ2.5 can be homozygous, heterozygous in *cis*, and heterozygous in *trans*. People carrying the DQ2.5 haplotype have a high risk to develop celiac disease, in comparison to those carrying DQ2.2 (15). DQB1^*^0201 is highly associated to disease risk; patients carrying two copies of DQB1^*^0201 show a five-fold risk to develop the disease compared to heterozygotes. The dosage of DQA1^*^0501 appears less tightly linked to the development of celiac disease. Thus, HLA-DQ2.5 homozygotes and HLA-DQ2.5*cis*/2.2 heterozygotes show a higher risk for CD, compared to HLA-DQ2.5*cis*/non-DQ2.2 heterozygotes (DQ2.5/X) and HLA-DQ2.5(*trans*) heterozygotes (15–18). Finally, the HLA-DQ8 genes associated with celiac disease, DQA1^*^0301 and DQB1^*^0302, confer a low risk in comparison to DQ2 genes (16). However, risk grade for CD in DQ2.5/DQ8 carriers is unclear, as some studies have classified these patients as having the highest genetic predisposition (16) with others as being intermediate risk only (19). Overall, about 35% of the genetic risk of developing celiac disease is associated with the presence of the HLA-DQ2/DQ8 haplotype (20, 21).

To further complicate the subject, HLA-DQ haplotypes are linked to HLA-DR genes, forming HLA-DR-DQ haplotypes, mainly DR3-DQ2.5, DR5/7-DQ2.2, and DR4-DQ8 in the case of celiac disease. HLA-DR haplotypes are known to confer risk for a variety of immune-mediated disorders (Figure 1): HLA DR3 is associated to Graves disease, myasthenia gravis, systemic lupus erythematosus (SLE), Hashimoto's thyroiditis, and type 1 diabetes, the last two also associated to DR4. HLA-DR4 is also associated to rheumatoid arthritis (22). Risk can be independently conferred by DQ and DR alleles: for instance, type 1 diabetes risk is independently associated with both DQ2.5/8 alleles (HLA-DQB1^*^0201/HLA-DQB1^*^0302) and DR3/4 alleles (HLA-DRB1^*^0301/ HLA-DRB1^*^0405, HLA-DRB1^*^0401) (23). Increased risk for co-occurrence of type 1 diabetes and celiac disease has been reported for DR3-DQ2.5 homozygotes as well as for DR3-DQ2.5/DR4-DQ8 vs. celiac disease alone; on the contrary, DR3-DQ2.5/DR7-DQ2.2 did not increase the risk for co-occurrence (24).

Different studies have shown that the risk of developing celiac disease among the patient's relatives is higher than that of the general population. The prevalence of the disease in first-degree relatives (FDRs) is 7.5% and varies considerably depending on the degree of kinship (e.g., sisters and daughters have higher risk than fathers and sons) (25). These potential patients should be monitored and tested periodically for anti-TG2 and anti-endomysial antibodies. However, as mentioned above, only those carrying the HLA-DQ2/DQ8 can develop the disease; therefore, FDRs are preliminary stratified for the presence of these haplotypes (Figure 1). This genetic typing is generally based on the amplification of genomic DNA, extracted from blood samples or mouth swabs, by polymerase chain reaction (PCR) followed by hybridization of the PCR product with sequence-specific oligonucleotide probes (26). An alternative approach is the direct detection of haplotypes by real-time PCR (27).

Due to the high complexity of the HLA locus, only recently, through a fine mapping analysis, it was discovered that other portions of HLA besides DQ2/DQ8 are important in celiac disease (28, 29): specifically, the small nucleotide polymorphism (SNPs) rs2301226 located within the HLA class II locus and connected by expression quantitative trait loci (eQTL) to the expression HLA-DPB1 and B3GALT4; the HLA class I alleles HLA-B0801 and HLA-B3906 as well as the SNPs that influence the expression of HLA-F have all been associated to celiac disease (28). Excluding the HLA-DQ2/DQ8, genetic information within the HLA locus represents a further 18% of the risk (28).

## Genetic Variants of NON-HLA Loci Associated With Celiac Disease

Although robust evidence on the role of HLA molecules and particular genotypes may explain their role in celiac disease pathogenesis, these are important yet not sufficient factors to explain the occurrence of the disease in ~3% of all individuals harboring DQ2 or DQ8 HLA loci. Sequencing of the human genome opened the possibility of mapping genetic variants and analyzing their association with complex diseases. To date, over 40 additional genetic loci have been associated with celiac disease outside the HLA region (30). These variants are mainly SNPs found in non-coding regions, often within enhancers, suggesting an effect on gene expression rather than causing modifications of protein functions. The pathogenic mechanisms linking celiac disease with the few genetic variants found within encoding regions (e.g., MMEL1, SH2B3, IRAK1, and NCF2) are not fully understood, although these proteins play important roles in adaptive immune response, immune cell signaling, T cell maturation, and differentiation (29). Association of HLA and Non-HLA alleles may favor the occurrence of celiac disease as shown in a large population study of 2,308 cases and 4,585 controls, in which risk increased six-fold when 13 non-HLA alleles were present, compared to those with zero to five alleles (31). Non-HLA alleles may also ameliorate risk prediction. In a study involving 2,675 cases and 2,815 controls, adding 57 non-HLA variants to HLA testing showed a statistically significant improvement of the receiver operating characteristic (ROC) curve from 0.823 (for HLA only) to 0.854, which translates in a reclassification of risk in 11% of individuals (32). In a similar approach, Izzo et al. improved the prediction of the disease in a cohort of first-degree relatives, particularly in the low HLA risk groups (33). The association of such non-HLA regions with celiac disease may show some geographic variation, being statistically significant in Swedish children but insignificant in US and Finland (34). However, the possible use of non-HLA variants for the stratification of celiac patients has not yet been introduced in the clinical setting (Figure 1). A recent study selected 42 SNPs from a European case control study of about 12,000 patients and as many controls and showed that these SNPs can discriminate celiac disease more than HLA-DQ2/DQ8 in both adults (1,280 cases) and children (114 cases) cohorts (35). Moreover, since clinical presentation of celiac disease differs in siblings with similar HLA haplotypes, it has been suggested that non-HLA genes may play a significant role more so than HLA-concordance in siblings (36). It has also been reported that risk variants in the IL2-IL21 region may further contribute to celiac disease susceptibility (37). The same study group proposed seven other regions selected from 1,164 non-HLA SNPs, which may modulate innate and adaptive immunity (20). Others have demonstrated the presence of non-HLA risk variants that act by influencing regulatory regions consistent with those previously reported in other immune-mediated diseases (29).

These observations may be relevant also to the occurrence of autoimmune disorders in celiac disease. In fact, celiac disease may present with a number of associated autoimmune conditions, and some of these, namely, multiple sclerosis (MS), autoimmune thyroiditis, psoriasis, vitiligo, alopecia, inflammatory bowel disease (IBD), autoimmune hepatitis, primary biliary cholangitis, type I diabetes, rheumatoid arthritis (RA), ankylosing spondylitis, and systemic lupus erythematosus (SLE) show some genetic overlap with celiac disease; in particular, IBD, MS, and RA show a substantial number of associated loci (110, 97, and 101, respectively) (38).

Several studies have observed a shared genetic pathway for non-HLA genes that may predispose to autoimmune diseases in children and adults (39–43). Although relevant to its occurrence, non-HLA loci do not appear to favor progression of the disease toward severe complications such as refractory celiac disease type II (RCD II), a condition that frequently evolves to intestinal lymphoma (44). In a 2018 study, none out of 39 known non-HLA genetic loci associated to celiac disease were found to be significantly associated with susceptibility to RCD II.

Finally, intestinal biopsies and immune cells eQTL analysis, aimed at understanding the possible functional role of the SNPs, led to the identification of over 100 candidate genes. Integrated analysis of data related to genetic regulome elements suggested that SNPs associated with celiac disease mainly impact on the regulatory layer of the cells. For example, over 75% of the SNPs belong to DNAse I hypersensitive regions and might influence the binding of numerous transcription factors (30).

## Gene Expression Altered in the Small Intestine of Patients Affected by Celiac Disease

Genome-wide gene expression studies were also used to further our understanding of celiac disease. These studies, using intestinal biopsies, revealed a number of differentially expressed genes (DEG) including APOC3, CYP3A4, OCLN, MAD2L1, MKI67, CXCL11, and IL17A among the most representative (45). A more recent study identified over 1,000 DEGs; interestingly, of the 79 genes potentially involved in celiac disease by the genetic mapping of non-HLA loci (30), 65 were among the DEGs of this study (46). Pathway analysis suggested that these DEGs are involved in carbohydrate, lipid, and amino acid metabolism, microorganisms infection, native and adaptive immune response, intestinal permeability, and autoimmune conditions. Twenty-seven DEGs following validation by qPCR were investigated by principal component analysis (PCA). The PC1 summarizing 79% of gene expression variance strongly correlated with the severity of mucosal damage. It is interesting to note that five genes (CXCL10, GBP5, IFI27, IFNG, and UBD) were differentially expressed in patients with potential celiac disease at the time of the investigation and who developed overt celiac condition afterwards (46).

## Clinical Presentation of NON-HLA Gluten-Related Disorders

Non-HLA gluten-related disorders share similar clinical characteristics with irritable bowel syndrome (IBS). Indeed, some studies have included this condition in the IBS spectrum because of their overlapping symptoms. In this scenario, fermentable oligo-, di-, and monosaccharides and polyols (FODMAPs) could have a critical role in driving the pathogenesis and triggering symptoms (47). Conversely, other authors depicted a key ability of gluten alone in promoting the clinical aspects in specific clusters of patients (47–50).

Overall, patients with non-HLA gluten-related disorders usually report IBS-like gastrointestinal symptoms. Clinical manifestations often occur a short period after gluten assumption and can last from hours to few days (50). Abdominal pain and bloating, altered bowel habits compatible with IBS-D (diarrhea), IBS-C (constipation) or IBS-M (mixed), nausea, and reflux are commonly described (51, 52). Moreover, some subjects can also report extra-intestinal manifestations such as headache, fatigue, foggy mind, fibro-myalgia, anxiety, depression, altered sleep pattern, weight gain, joint pain, skin rash, and dermatitis (48, 51–54). These manifestations can also be described in celiac disease and are not connected to FODMAPs mechanisms of action. Thus, they could mainly represent a potential specific effect of gluten (50). Similarly, skin manifestations are often described in an important percentage of subjects and improve when on a gluten-free diet (55, 56).

Finally, a potential relationship between non-HLA gluten-related disorders and neuropsychiatric diseases has been suggested. In particular, autism spectrum and schizophrenia have been mainly involved (50). This connection may be due to the increased intestinal permeability (i.e., leaky-gut syndrome) often described in non-HLA gluten-related disorders. In this context, gluten and gluten-related molecules can pass into the bloodstream along the gut–brain axis and have a strong impact on the endogenous opioid and neurotransmission brain system (57, 58). Gluten molecules could have the ability to interact with opioid receptors, cause immune cells activation, trigger neuro-inflammation, and overall modulate patients' behavior. Furthermore, gluten may affect serotonin production and thus play a potential role in depression spectrum disorders. Although the exact mechanisms remain unclear, a possible rationale could be linked to gut microbiota activity, a major direct and indirect source of serotonin, which could be negatively influenced by gluten-containing foods (50).

## Gene and Microrna Expression is Dysregulated in the Small Intestine of Patients Affected by NCWS

In contrast with celiac disease, very little is known about the genetics of NCWS. First of all, patients affected by NCWS do not carry any characteristic HLA (59). HLA DQ2 and DQ8 are present in about 40–50% of NCWS patients, a percentage slightly above the prevalence of these haplotypes in the general population which suggests that DQ2/DQ8 has minor relevance, if any, in the development of NCWS (11). Furthermore, genotyping of NCWS is not yet available. The examination of a few genes involved in the immune response demonstrated the downregulation of the T-regulator marker FOXP3 and the upregulation of Toll like receptor 2 suggesting a possible role of innate immunity in NCWS. Claudin was also upregulated, in agreement with the increase of intestinal barrier function (12).

In a recent study by our laboratory we identified seven miRNA (hsa-miR-19b-3p, hsa-miR-19a-3p, hsa-miR-186-5p, hsa-miR-17-5p, hsa-miR-145-5p, hsa-miR-30e-5p, and hsa-miR-143-3p) whose expression was higher in the intestinal mucosa of NCWS patients than in controls with dyspeptic symptoms or celiac disease (60). Remarkably, six of these miRNA were also dysregulated in peripheral blood leukocytes of NCWS. In addition, from an analysis of gene expression on a genomic scale in intestinal biopsies of NCWS patients, we have identified 300 DEGs (61). The vast majority of these transcripts were long non-coding RNA (lncRNA), while only 36% were protein-coding transcripts. Although still poorly investigated, lncRNA are considered regulatory RNAs involved in various pathophysiological functions including the immune response and inflammation (62–64). Similarly to celiac disease, non-coding regions of the genome might be important also for the development of NCWS. These lncRNAs can exert their effects by controlling the organization of chromatin, the transcription, the splicing, the stability of mRNAs, or the availability of miRNAs (65). In fact, further investigation is needed to understand their role in NCWS. Functional analysis of the coding transcripts revealed the presence of genes of inflammation, innate immunity, or previously associated with autoimmune diseases. Ingenuity pathway analysis suggested that hedgehog signaling pathway and regulators of circadian rhythm may also have a role in NCWS (61). Finally, these biomarkers (miRNAs and lncRNAs) after appropriate validation in a clinical setting could be potentially useful for the diagnosis of NCWS (Figure 1).

## Conclusions

Identification and characterization of non-HLA celiac disease genes is an important tool to the clarification of the pathophysiology of celiac disease as well as for risk prediction and diagnosis. It may also help identify patients with a borderline disease or with discordant clinical, laboratory, and pathology features. The risk of developing extraintestinal manifestations of the disease or the progression toward more severe stages of celiac disease might also be predicted. Finally, disentangling the biologic role of non-HLA genes may clarify the disease process and enlighten the road to potential new treatments.

## Author Contributions

MS and MN conceived the manuscript and contributed to its organization, creation of a draft, revision, and editing. LL and KE contributed to the writing and editing of the manuscript, as well as the literature revision. All authors contributed to the article and approved the submitted version.

## Conflict of Interest

The authors declare that the research was conducted in the absence of any commercial or financial relationships that could be construed as a potential conflict of interest.
